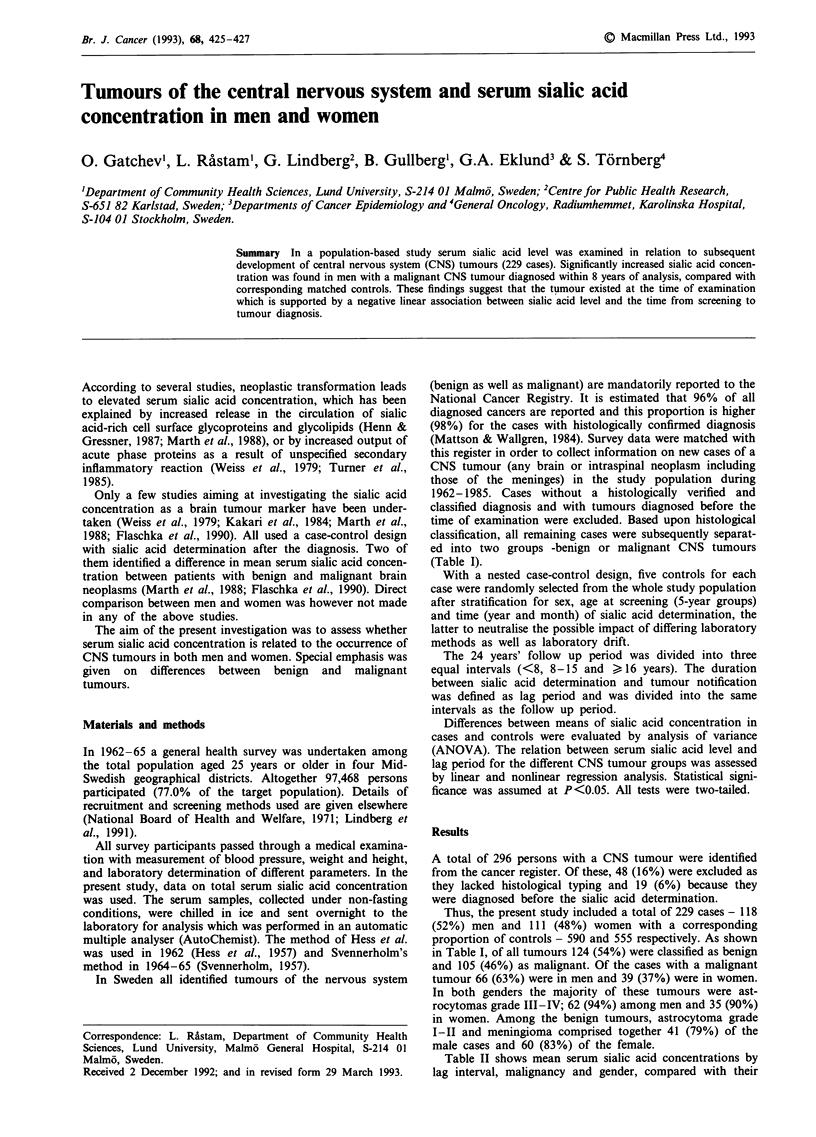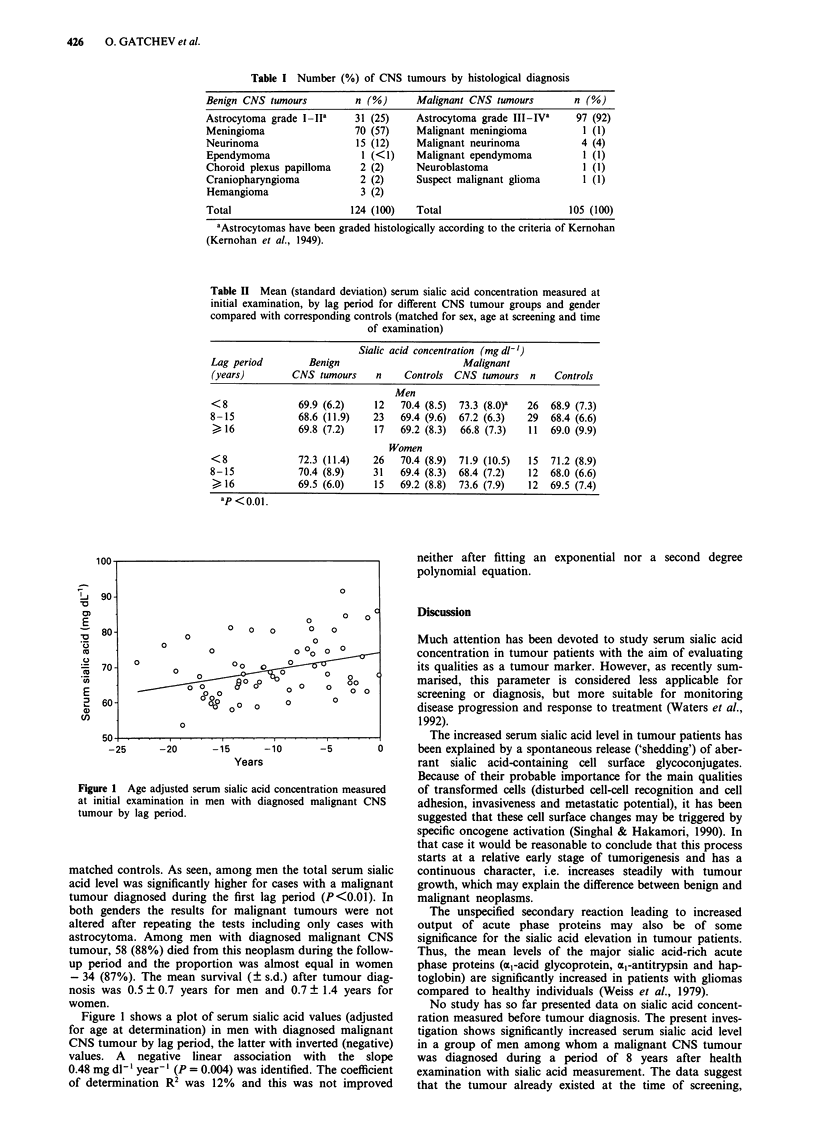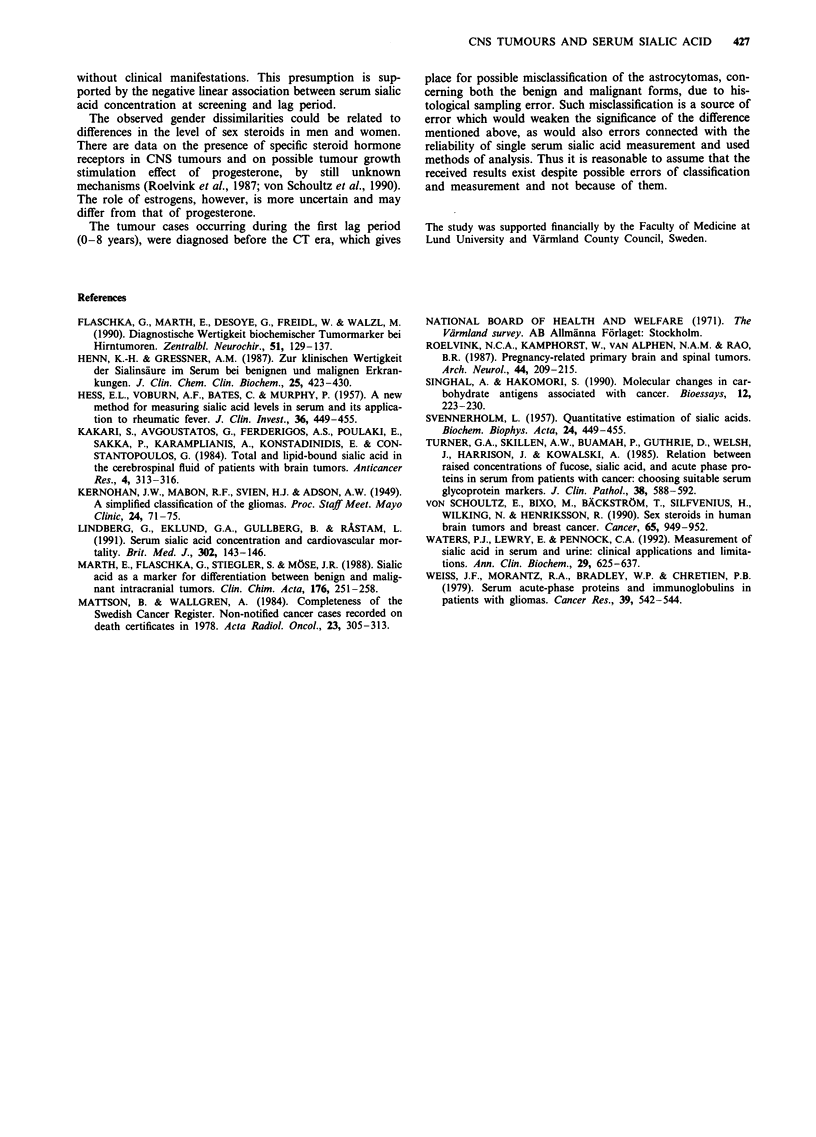# Tumours of the central nervous system and serum sialic acid concentration in men and women.

**DOI:** 10.1038/bjc.1993.353

**Published:** 1993-08

**Authors:** O. Gatchev, L. Råstam, G. Lindberg, B. Gullberg, G. A. Eklund, S. Törnberg

**Affiliations:** Department of Community Health Sciences, Lund University, Malmö, Sweden.

## Abstract

In a population-based study serum sialic acid level was examined in relation to subsequent development of central nervous system (CNS) tumours (229 cases). Significantly increased sialic acid concentration was found in men with a malignant CNS tumour diagnosed within 8 years of analysis, compared with corresponding matched controls. These findings suggest that the tumour existed at the time of examination which is supported by a negative linear association between sialic acid level and the time from screening to tumour diagnosis.


Br. J. Cancer (1993), 68, 425–427 © Macmillan Press Ltd., 1993

### Tumours of the central nervous system and serum sialic acid concentration in men and women

O. Gatchev[1], L. Råstam[1], G. Lindberg[2], B. Gullberg[1], G.A. Eklund[3] & S. Törnberg[4]

[1]*Department of Community Health Sciences, Lund University, S-214 01 Malmö, Sweden;* [2]*Centre for Public Health Research, S-651 82 Karlstad, Sweden;* [3]*Departments of Cancer Epidemiology and* [4]*General Oncology, Radiumhemmet, Karolinska Hospital, S-104 01 Stockholm, Sweden.*

**Summary** In a population-based study serum sialic acid level was examined in relation to subsequent development of central nervous system (CNS) tumours (229 cases). Significantly increased sialic acid concentration was found in men with a malignant CNS tumour diagnosed within 8 years of analysis, compared with corresponding matched controls. These findings suggest that the tumour existed at the time of examination which is supported by a negative linear association between sialic acid level and the time from screening to tumour diagnosis.

According to several studies, neoplastic transformation leads to elevated serum sialic acid concentration, which has been explained by increased release in the circulation of sialic acid-rich cell surface glycoproteins and glycolipids (Henn & Gressner, 1987; Marth *et al.*, 1988), or by increased output of acute phase proteins as a result of unspecified secondary inflammatory reaction (Weiss *et al.*, 1979; Turner *et al.*, 1985).

Only a few studies aiming at investigating the sialic acid concentration as a brain tumour marker have been undertaken (Weiss *et al.*, 1979; Kakari *et al.*, 1984; Marth *et al.*, 1988; Flaschka *et al.*, 1990). All used a case-control design with sialic acid determination after the diagnosis. Two of them identified a difference in mean serum sialic acid concentration between patients with benign and malignant brain neoplasms (Marth *et al.*, 1988; Flaschka *et al.*, 1990). Direct comparison between men and women was however not made in any of the above studies.

The aim of the present investigation was to assess whether serum sialic acid concentration is related to the occurrence of CNS tumours in both men and women. Special emphasis was given on differences between benign and malignant tumours.

### Materials and methods

In 1962–65 a general health survey was undertaken among the total population aged 25 years or older in four Mid-Swedish geographical districts. Altogether 97,468 persons participated (77.0% of the target population). Details of recruitment and screening methods used are given elsewhere (National Board of Health and Welfare, 1971; Lindberg *et al.*, 1991).

All survey participants passed through a medical examination with measurement of blood pressure, weight and height, and laboratory determination of different parameters. In the present study, data on total serum sialic acid concentration was used. The serum samples, collected under non-fasting conditions, were chilled in ice and sent overnight to the laboratory for analysis which was performed in an automatic multiple analyser (AutoChemist). The method of Hess *et al.* was used in 1962 (Hess *et al.*, 1957) and Svennerholm's method in 1964–65 (Svennerholm, 1957).

In Sweden all identified tumours of the nervous system (benign as well as malignant) are mandatorily reported to the National Cancer Registry. It is estimated that 96% of all diagnosed cancers are reported and this proportion is higher (98%) for the cases with histologically confirmed diagnosis (Mattson & Wallgren, 1984). Survey data were matched with this register in order to collect information on new cases of a CNS tumour (any brain or intraspinal neoplasm including those of the meninges) in the study population during 1962–1985. Cases without a histologically verified and classified diagnosis and with tumours diagnosed before the time of examination were excluded. Based upon histological classification, all remaining cases were subsequently separated into two groups -benign or malignant CNS tumours (Table I).

With a nested case-control design, five controls for each case were randomly selected from the whole study population after stratification for sex, age at screening (5-year groups) and time (year and month) of sialic acid determination, the latter to neutralise the possible impact of differing laboratory methods as well as laboratory drift.

The 24 years' follow up period was divided into three equal intervals (<8, 8–15 and ⩾16 years). The duration between sialic acid determination and tumour notification was defined as lag period and was divided into the same intervals as the follow up period.

Differences between means of sialic acid concentration in cases and controls were evaluated by analysis of variance (ANOVA). The relation between serum sialic acid level and lag period for the different CNS tumour groups was assessed by linear and nonlinear regression analysis. Statistical significance was assumed at $P<0.05$. All tests were two-tailed.

### Results

A total of 296 persons with a CNS tumour were identified from the cancer register. Of these, 48 (16%) were excluded as they lacked histological typing and 19 (6%) because they were diagnosed before the sialic acid determination.

Thus, the present study included a total of 229 cases – 118 (52%) men and 111 (48%) women with a corresponding proportion of controls – 590 and 555 respectively. As shown in Table I, of all tumours 124 (54%) were classified as benign and 105 (46%) as malignant. Of the cases with a malignant tumour 66 (63%) were in men and 39 (37%) were in women. In both genders the majority of these tumours were astrocytomas grade III–IV; 62 (94%) among men and 35 (90%) in women. Among the benign tumours, astrocytoma grade I–II and meningioma comprised together 41 (79%) of the male cases and 60 (83%) of the female.

Table II shows mean serum sialic acid concentrations by lag interval, malignancy and gender, compared with their

Correspondence: L. Råstam, Department of Community Health Sciences, Lund University, Malmö General Hospital, S-214 01 Malmö, Sweden.
Received 2 December 1992; and in revised form 29 March 1993.

**Table I** Number (%) of CNS tumours by histological diagnosis

| *Benign CNS tumours* | *n (%)* | *Malignant CNS tumours* | *n (%)* |
|---|---|---|---|
| Astrocytoma grade I–II[a] | 31 (25) | Astrocytoma grade III–IV[a] | 97 (92) |
| Meningioma | 70 (57) | Malignant meningioma | 1 (1) |
| Neurinoma | 15 (12) | Malignant neurinoma | 4 (4) |
| Ependymoma | 1 (<1) | Malignant ependymoma | 1 (1) |
| Choroid plexus papilloma | 2 (2) | Neuroblastoma | 1 (1) |
| Craniopharyngioma | 2 (2) | Suspect malignant glioma | 1 (1) |
| Hemangioma | 3 (2) | | |
| Total | 124 (100) | Total | 105 (100) |

[a]Astrocytomas have been graded histologically according to the criteria of Kernohan (Kernohan *et al.*, 1949).

**Table II** Mean (standard deviation) serum sialic acid concentration measured at initial examination, by lag period for different CNS tumour groups and gender compared with corresponding controls (matched for sex, age at screening and time of examination)

| | *Sialic acid concentration (mg dl$^{-1}$)* | | | | | |
|---|---|---|---|---|---|---|
| *Lag period (years)* | *Benign CNS tumours* | *n* | *Controls* | *Malignant CNS tumours* | *n* | *Controls* |
| | | | *Men* | | | |
| <8 | 69.9 (6.2) | 12 | 70.4 (8.5) | 73.3 (8.0)[a] | 26 | 68.9 (7.3) |
| 8–15 | 68.6 (11.9) | 23 | 69.4 (9.6) | 67.2 (6.3) | 29 | 68.4 (6.6) |
| ≥16 | 69.8 (7.2) | 17 | 69.2 (8.3) | 66.8 (7.3) | 11 | 69.0 (9.9) |
| | | | *Women* | | | |
| <8 | 72.3 (11.4) | 26 | 70.4 (8.9) | 71.9 (10.5) | 15 | 71.2 (8.9) |
| 8–15 | 70.4 (8.9) | 31 | 69.4 (8.3) | 68.4 (7.2) | 12 | 68.0 (6.6) |
| ≥16 | 69.5 (6.0) | 15 | 69.2 (8.8) | 73.6 (7.9) | 12 | 69.5 (7.4) |

[a]$P < 0.01$.

**Figure 1** Age adjusted serum sialic acid concentration measured at initial examination in men with diagnosed malignant CNS tumour by lag period.

matched controls. As seen, among men the total serum sialic acid level was significantly higher for cases with a malignant tumour diagnosed during the first lag period ($P<0.01$). In both genders the results for malignant tumours were not altered after repeating the tests including only cases with astrocytoma. Among men with diagnosed malignant CNS tumour, 58 (88%) died from this neoplasm during the follow-up period and the proportion was almost equal in women – 34 (87%). The mean survival (± s.d.) after tumour diagnosis was $0.5 \pm 0.7$ years for men and $0.7 \pm 1.4$ years for women.

Figure 1 shows a plot of serum sialic acid values (adjusted for age at determination) in men with diagnosed malignant CNS tumour by lag period, the latter with inverted (negative) values. A negative linear association with the slope 0.48 mg dl$^{-1}$ year$^{-1}$ ($P = 0.004$) was identified. The coefficient of determination $R^2$ was 12% and this was not improved neither after fitting an exponential nor a second degree polynomial equation.

### Discussion

Much attention has been devoted to study serum sialic acid concentration in tumour patients with the aim of evaluating its qualities as a tumour marker. However, as recently summarised, this parameter is considered less applicable for screening or diagnosis, but more suitable for monitoring disease progression and response to treatment (Waters *et al.*, 1992).

The increased serum sialic acid level in tumour patients has been explained by a spontaneous release ('shedding') of aberrant sialic acid-containing cell surface glycoconjugates. Because of their probable importance for the main qualities of transformed cells (disturbed cell-cell recognition and cell adhesion, invasiveness and metastatic potential), it has been suggested that these cell surface changes may be triggered by specific oncogene activation (Singhal & Hakamori, 1990). In that case it would be reasonable to conclude that this process starts at a relative early stage of tumorigenesis and has a continuous character, i.e. increases steadily with tumour growth, which may explain the difference between benign and malignant neoplasms.

The unspecified secondary reaction leading to increased output of acute phase proteins may also be of some significance for the sialic acid elevation in tumour patients. Thus, the mean levels of the major sialic acid-rich acute phase proteins ($\alpha_1$-acid glycoprotein, $\alpha_1$-antitrypsin and haptoglobin) are significantly increased in patients with gliomas compared to healthy individuals (Weiss *et al.*, 1979).

No study has so far presented data on sialic acid concentration measured before tumour diagnosis. The present investigation shows significantly increased serum sialic acid level in a group of men among whom a malignant CNS tumour was diagnosed during a period of 8 years after health examination with sialic acid measurement. The data suggest that the tumour already existed at the time of screening, without clinical manifestations. This presumption is supported by the negative linear association between serum sialic acid concentration at screening and lag period.

The observed gender dissimilarities could be related to differences in the level of sex steroids in men and women. There are data on the presence of specific steroid hormone receptors in CNS tumours and on possible tumour growth stimulation effect of progesterone, by still unknown mechanisms (Roelvink *et al.*, 1987; von Schoultz *et al.*, 1990). The role of estrogens, however, is more uncertain and may differ from that of progesterone.

The tumour cases occurring during the first lag period (0–8 years), were diagnosed before the CT era, which gives place for possible misclassification of the astrocytomas, concerning both the benign and malignant forms, due to histological sampling error. Such misclassification is a source of error which would weaken the significance of the difference mentioned above, as would also errors connected with the reliability of single serum sialic acid measurement and used methods of analysis. Thus it is reasonable to assume that the received results exist despite possible errors of classification and measurement and not because of them.

The study was supported financially by the Faculty of Medicine at Lund University and Värmland County Council, Sweden.

### References

FLASCHKA, G., MARTH, E., DESOYE, G., FREIDL, W. & WALZL, M. (1990). Diagnostische Wertigkeit biochemischer Tumormarker bei Hirntumoren. *Zentralbl. Neurochir.*, **51**, 129–137.

HENN, K.-H. & GRESSNER, A.M. (1987). Zur klinischen Wertigkeit der Sialinsäure im Serum bei benignen und malignen Erkrankungen. *J. Clin. Chem. Clin. Biochem.*, **25**, 423–430.

HESS, E.L., VOBURN, A.F., BATES, C. & MURPHY, P. (1957). A new method for measuring sialic acid levels in serum and its application to rheumatic fever. *J. Clin. Invest.*, **36**, 449–455.

KAKARI, S., AVGOUSTATOS, G., FERDERIGOS, A.S., POULAKI, E., SAKKA, P., KARAMPLIANIS, A., KONSTADINIDIS, E. & CONSTANTOPOULOS, G. (1984). Total and lipid-bound sialic acid in the cerebrospinal fluid of patients with brain tumors. *Anticancer Res.*, **4**, 313–316.

KERNOHAN, J.W., MABON, R.F., SVIEN, H.J. & ADSON, A.W. (1949). A simplified classification of the gliomas. *Proc. Staff Meet. Mayo Clinic*, **24**, 71–75.

LINDBERG, G., EKLUND, G.A., GULLBERG, B. & RÅSTAM, L. (1991). Serum sialic acid concentration and cardiovascular mortality. *Brit. Med. J.*, **302**, 143–146.

MARTH, E., FLASCHKA, G., STIEGLER, S. & MÖSE, J.R. (1988). Sialic acid as a marker for differentiation between benign and malignant intracranial tumors. *Clin. Chim. Acta*, **176**, 251–258.

MATTSON, B. & WALLGREN, A. (1984). Completeness of the Swedish Cancer Register. Non-notified cancer cases recorded on death certificates in 1978. *Acta Radiol. Oncol.*, **23**, 305–313.

NATIONAL BOARD OF HEALTH AND WELFARE (1971). *The Värmland survey.* AB Allmänna Förlaget: Stockholm.

ROELVINK, N.C.A., KAMPHORST, W., VAN ALPHEN, N.A.M. & RAO, B.R. (1987). Pregnancy-related primary brain and spinal tumors. *Arch. Neurol.*, **44**, 209–215.

SINGHAL, A. & HAKOMORI, S. (1990). Molecular changes in carbohydrate antigens associated with cancer. *Bioessays*, **12**, 223–230.

SVENNERHOLM, L. (1957). Quantitative estimation of sialic acids. *Biochem. Biophys. Acta*, **24**, 449–455.

TURNER, G.A., SKILLEN, A.W., BUAMAH, P., GUTHRIE, D., WELSH, J., HARRISON, J. & KOWALSKI, A. (1985). Relation between raised concentrations of fucose, sialic acid, and acute phase proteins in serum from patients with cancer: choosing suitable serum glycoprotein markers. *J. Clin. Pathol.*, **38**, 588–592.

VON SCHOULTZ, E., BIXO, M., BÄCKSTRÖM, T., SILFVENIUS, H., WILKING, N. & HENRIKSSON, R. (1990). Sex steroids in human brain tumors and breast cancer. *Cancer*, **65**, 949–952.

WATERS, P.J., LEWRY, E. & PENNOCK, C.A. (1992). Measurement of sialic acid in serum and urine: clinical applications and limitations. *Ann. Clin. Biochem.*, **29**, 625–637.

WEISS, J.F., MORANTZ, R.A., BRADLEY, W.P. & CHRETIEN, P.B. (1979). Serum acute-phase proteins and immunoglobulins in patients with gliomas. *Cancer Res.*, **39**, 542–544.